# Synthesis and biological evaluation of novel quinolone derivatives dual targeting histone deacetylase and tubulin polymerization as antiproliferative agents†

**DOI:** 10.1039/c8ra02578a

**Published:** 2018-05-04

**Authors:** Xuan Wang, Xiaoye Jiang, Shiyou Sun, Yongqiong Liu

**Affiliations:** City College, Wuhan University of Science and Technology Wuhan 430000 China xwang887@126.com +86-2786467906

## Abstract

A strategy to develop chemotherapy agents by combining two complimentary chemo-active groups into a single molecule may have higher efficacy and fewer side effects than that of single-target drugs. In this article, we describe the synthesis and evaluation of a series of novel dual-acting levofloxacin–HDACi conjugates to target both histone deacetylase (HDAC) and tubulin polymerization. These bifunctional conjugates exhibited potent inhibitory activities against HDACs and tubulin polymerization. In docking analysis provides a structural basis for HDACs inhibition activities. Moreover, these conjugates showed selective anticancer activity that is more potent against MCF-7 compared to other four cancer cells A549, HepG2, PC-3, HeLa, but they had no toxicity toward normal cells.

## Abstract 2

Synthesis of a series of novel dual-acting levofloxacin–HDACi conjugates, which show potent inhibitory activities against HDACs, tubulin polymerization, and significant antiproliferative effect on MCF-7 cells.

## Graphical abstract

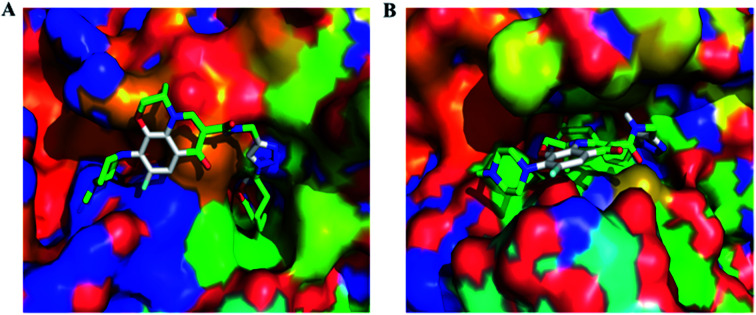

Cancer is a highly complex multifactorial disease involving multiple cross-talking between signaling networks. Almost all single-target-based drugs suffer from severe toxicities or other undesirable side effects. In contrast, combination therapy, which combines multiple anticancer agents working with different mechanisms, might have superior efficacy and fewer side effects compared to single-target treatments.^1,2^

Histone deacetylases (HDACs) are epigenetic enzymes that are capable of removing acetyl groups from ε-amino groups of lysine residues in histone or other nonhistone proteins.^3^ Abnormal expression of HDACs has been observed in various types of cancer,^4–6^ and these enzymes have emerged as important targets in the development of anticancer drugs. Consequently, inhibition of HDAC activity is now recognized as a powerful strategy for cancer therapy. There are 18 human HDAC isoforms categorized into four major classes: class I (HDACs 1, 2, 3, 8), class IIa (HDACS 4, 5, 7, 9), class IIb (HDACs 6 and 10), and class IV (HDAC 11) are Zn^2+^-dependent metalloenzymes, while class III (SirTs 1–7) are NDA^+^-dependent sirtuins.^7,8^ Of these HDAC isoforms, only HDACs 1, 2, 3, and 6 have shown biologically relevant deacetylation ability.^9^ Specially, selective inhibition of HDAC6 may have fewer side effects than pan-HDAC and class I isoform.^10–12^

To date, more than twenty HDAC inhibitors have been initiated in clinical trials, and four HDAC inhibitors vorinostat (SAHA),^13^ romidepsin (FK-228),^14^ belinostat (PXD-101),^15,16^ panobinostat (LBH-589),^17^ have been approved by FDA for the treatment of T-cell lymphoma, cutaneous T-cell lymphoma and multiple myeloma. However, most of them are pan-HDAC (SAHA, LBH-589) or class I selective (FK-228, PXD-101) inhibitors, which usually lead to several mild to severe side effects.^16,18,19^ In addition, most of HDAC inhibitors lack visible efficacy against solid tumor,^14,20^ the doses given in clinical are much higher, which severely limit their clinical utility for the treatment of broad spectrum of cancer. Therefore, preclinical evaluation of new HDAC inhibitors will need to focus on improving HDAC isoform selectivity and enhancing potency against solid tumors. One strategy may be able to ameliorate the shortcomings of current inhibitors, which is to develop a dual-acting HDAC inhibitor (HDACi) by incorporation of the surface recognition group of prototypical HDACi into other anticancer drugs, forming a single molecule that can modulate intracellular multiple targets, other than various HDAC isoforms. So far, a few examples of bifunctional HDACi-derived conjugates have been obtained.^21–26^ Expansion of the diversity of such bifunctional conjugates could lead to broad acting, therapeutically viable anticancer drugs.

In another aspect, fluoroquinolones (FQs) have recently been proven as an excellent class of broad-spectrum anticancer drugs against a variety of cancer cells such as bladder cancer,^27^ non-small cell lung carcinoma,^28^ colorectal carcinoma cells,^29^*etc.* For instance, it has been demonstrated that levofloxacin (Lv) displays antiproliferative activity against various cancer cells.^30,31^ Additionally, many of fluoroquinolones were potent inhibitors of tubulin polymerization and exhibited selective activity against some tumor cell types.^32,33^ More importantly, fluoroquinolones have favorable pharmacokinetic profiles and good adsorption, which possess an established record of safety.^34^ Therefore, on the basis of therapeutic effectives of aforementioned HDACi as well as fluoroquinolone, we conceived that concurrent inhibition of HDAC and tubulin polymerization would be a viable alternative approach for cancer treatment.

In this work, we describe the design, synthesis and biological evaluation of novel dual-action levofloxacin–HDACi conjugates, which can be prepared conveniently by direct connection of levofloxacin with a triazole-liked SAHA (Fig. 1). The levofloxacin–SAHA conjugates (compounds 8a–c and 9a–c) of this design not only have HDACi unit but also have a second pharmacologically quinolone scaffold. Thus, they expand the exploration of bifunctional HDACi-derived conjugates. For comparison, the carboxylic acid analogues (by replacing the hydroxamic acid (–CONHOH) group with (–COOH), compounds 6a–c and 7a–c) were also prepared and evaluated for their HDAC and tubulin polymerization inhibition activity, antiproliferative activity and cell-type selectivity, *etc.*

**Fig. 1:**
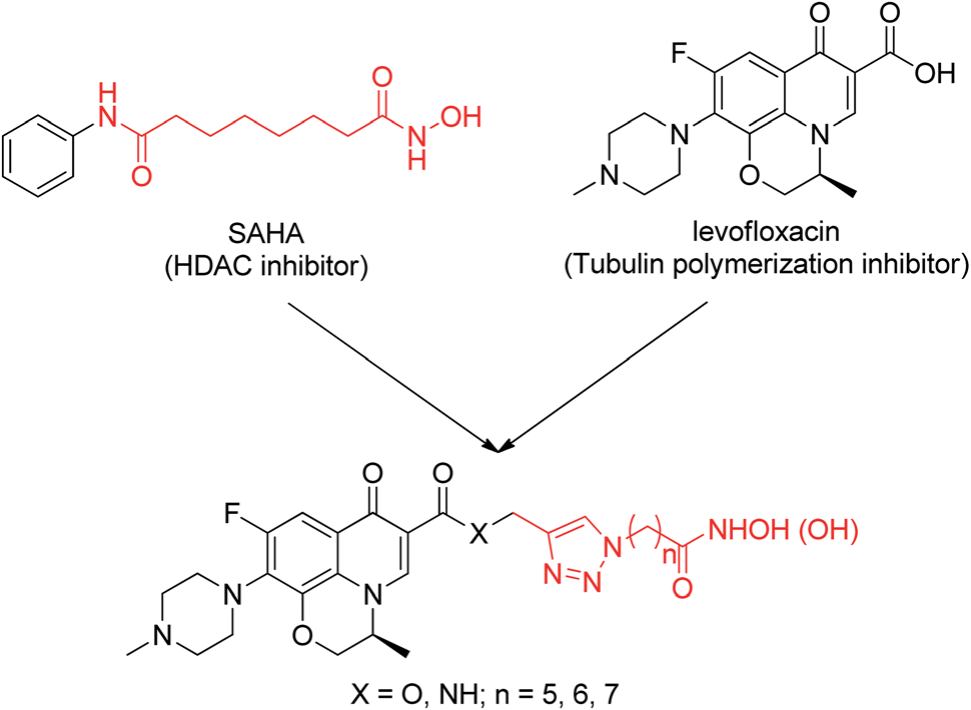
Design of dual-acting levofloxacin–HDACi conjugates.

## Results and discussion

### Chemical synthesis

**Scheme 1:**
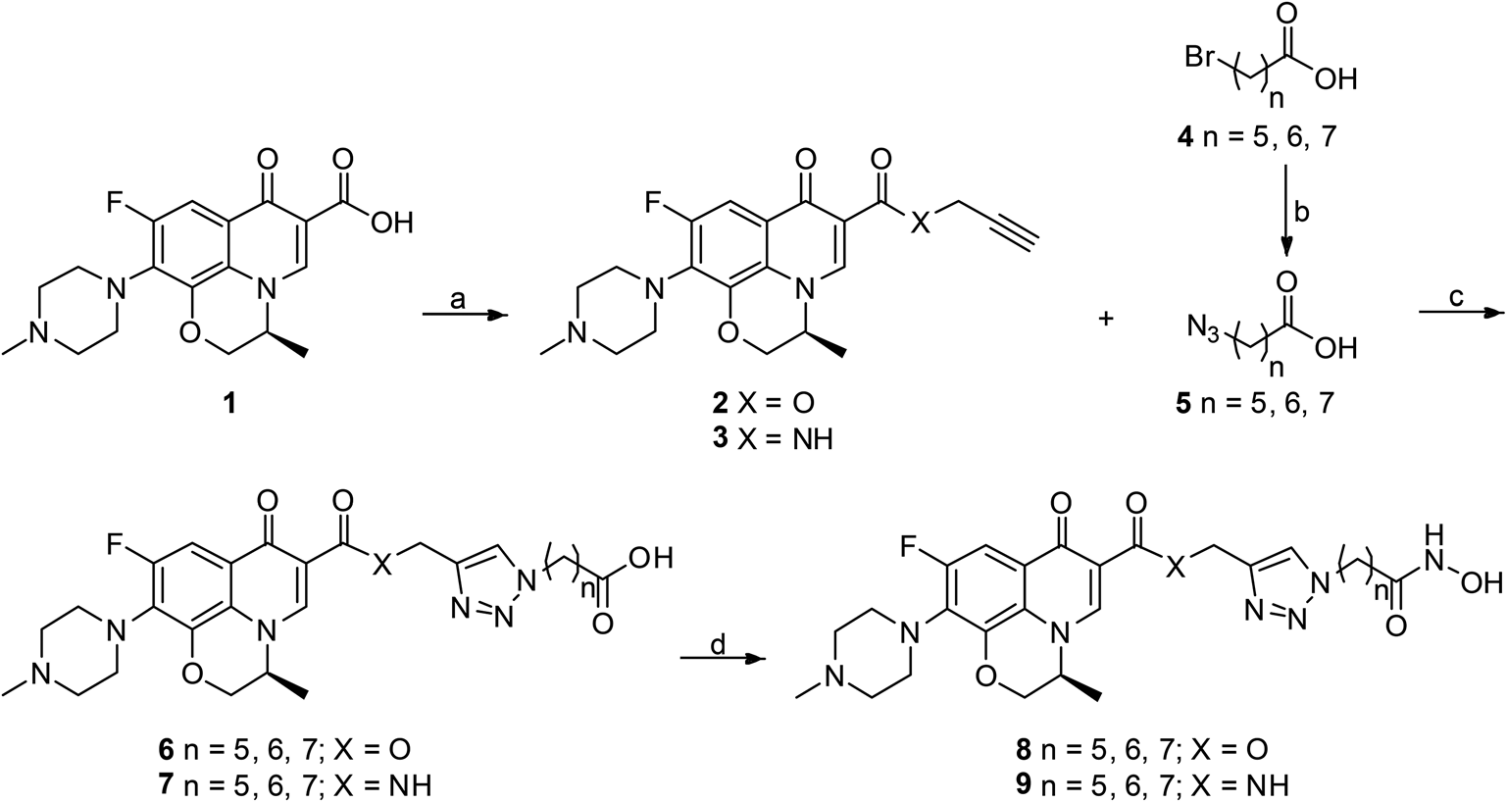
Synthesis of levofloxacin–HDACi conjugates. Reagents and conditions: (a) propargyl bromide, NaHCO_3_, DMF, 100 °C, 48 h for 2; TBTU, DIPEA, THF, rt, 5 h and then propargylamine, K_2_CO_3_, DMF, rt, 1 h for 3; (b) NaN_3_, DMF, 77 °C, 30 h; (c) CuSO_4_, Na-l-ascorbate, THF–H_2_O, rt, 20 h; (d) ClCO_2_Et, Et_3_N, THF, 0 °C, 15 min and then NH_2_OH·HCl, KOH, MeOH, rt, 1 h.

All designed levofloxacin–HDACi conjugations (6a–c, 7a–c, 8a–c, 9a–c) were achieved by the click chemistry as outlined in Scheme 1. In the initial step, treatment of levofloxacin 1 with propargyl bromide or propargylamine gave propargylated levofloxacin 2 and 3. On the other hand, important intermediate azidohexanoic acid 5 was prepared in one step from commercially available bromo alkyl acid 4. Subsequently, reaction of propargylated levofloxacin 2 or 3 with azidohexanoic acid 5 in the presence of CuSO_4_ and l-ascorbate afforded carboxylic acid conjugates 6–7. Finally, treatment of 6–7 with KOH/NH_2_OH in THF after activation of the carboxylate afforded hydroxamic acid conjugates 8–9.

### Conjugates exhibit potent HDAC inhibition activity

We tested all of levofloxacin–HDACi conjugates for HDAC inhibition activity against HDAC1, HDAC2 and HDAC6 isoforms, and SAHA as positive. The data was summarized in Table 1.

**Table 1:** IC_50_ values of conjugates for inhibition of HDAC1, HDAC2 and HDAC6

Compound	IC_50_ (μmol L^−1^)			Selectivity index	
	HDAC1	HDAC2	HDAC6	HDAC6/1	HDAC6/2
6a	8.2 ± 1.08	10.4 ± 1.87	2.1 ± 0.68	3.9	4.9
6b	5.7 ± 0.71	6.2 ± 0.92	1.4 ± 0.35	4.1	4.4
6c	6.9 ± 0.83	7.9 ± 0.76	4.7 ± 0.51	1.5	1.7
7a	4.6 ± 0.52	7.3 ± 0.93	1.9 ± 0.47	2.4	3.8
7b	2.1 ± 0.37	4.6 ± 0.62	1.1 ± 0.28	1.9	4.2
7c	3.8 ± 0.46	6.9 ± 0.55	2.7 ± 0.36	1.4	2.6
8a	0.075 ± 0.031	0.134 ± 0.049	0.043 ± 0.053	1.7	3.1
8b	0.067 ± 0.019	0.082 ± 0.015	0.030 ± 0.019	2.2	2.7
8c	0.104 ± 0.053	0.065 ± 0.016	0.052 ± 0.026	2.0	1.3
9a	0.073 ± 0.045	0.85 ± 0.057	0.049 ± 0.023	1.5	1.7
9b	0.029 ± 0.018	0.041 ± 0.014	0.021 ± 0.007	1.4	2.0
9c	0.038 ± 0.014	0.076 ± 0.022	0.041 ± 0.025	0.9	1.9
SAHA	0.044 ± 0.002	0.012 ± 0.003	0.036 ± 0.006	1.2	0.3

In general, these conjugates strongly inhibited HDAC1, HDAC2 and HDAC6 three isoforms. However, the zinc-binding group has very significant effects on HDAC inhibition activity of conjugates, and the hydroxamic acid conjugates 8–9 have superior HDAC inhibition activity relative to the carboxylic acid conjugates 6–7; actually, of the hydroxamic acid conjugates, 8–9 inhibit HDAC1, HDAC2 and HDAC6 with nanomolar range IC_50_s, while the carboxylic acid conjugates 6–7 was micromole, especially hydroxamic acid conjugate 9b (The dose–response curves are shown in Fig. S1†) showed the most potent anti-HDAC1 (IC_50_ = 29 nM) and HDAC6 (IC_50_ = 21 nM) activities; It was over 1.5-fold more potent than the SAHA in HDAC1 and HDAC6 inhibition (9b*vs.* SAHA). Additionally, the HDAC inhibition activity was very much dependent on the length of the HDACi unit of levofloxacin–HDACi conjugates. We observed that all conjugate sets show greatest inhibition activity with a linker length of 6 (6b, 7b, 8b, 9b, *n* = 6), however, either shorter (6a, 7a, 8a, 9a, *n* = 5) or longer chain lengths (6c, 7c, 8c, 9c, *n* = 7) result in reduced potency. Moreover, the connecting group of levofloxacin and HDACi unit, ester or amide group, also had effect on HDACs inhibition activity, and conjugates with an amide group were much potent than ester group containing conjugates. For example, when the amide group of 9c was replaced with an ester group (analogue 8c), it resulted in at least 2-fold reduced potency anti-HDAC1, 2 and 6.

### Tubulin polymerization inhibition

Some recent reports suggested that the antiproliferative activity of quinolinones resulted from the interaction with inhibition of tubulin polymerization.^35–37^ Thus, we also evaluated the abilities of levofloxacin–HDACi conjugates 6–9 in inhibiting the tubulin polymerization (Table 2).

**Table 2:** Effects of conjugates 6–9 on tubulin polymerization inhibition

Compound	IC_50_^a^ (μmol L^−1^)	Compound	IC_50_^a^ (μmol L^−1^)
6a	7.03 ± 0.74	8a	5.45 ± 1.18
6b	5.01 ± 0.25	8b	1.84 ± 0.77
6c	8.45 ± 0.76	8c	6.36 ± 0.24
7a	3.62 ± 0.51	9a	2.02 ± 0.44
7b	2.11 ± 0.33	9b	1.79 ± 0.21
7c	4.06 ± 1.42	9c	6.68 ± 0.28
Levofloxacin	11.27 ± 1.17	Colchicine	1.77 ± 0.14

a IC_50_ values are an average of three independent experiments ± standard deviation (mean ± SD).

In this assembly assay, conjugates 6–9 displayed potent inhibitory effects on tubulin polymerization with IC_50_ values ranging from 1.79 to 8.45 μM, and which were more active than parent compound levofloxacin (IC_50_ = 11.27 ± 1.17 μM). Specifically, conjugates 7b, 8b, 9a and 9b were even equipotent to reference compound CA-4, and the tubulin polymerization time-course plots and dose–response curves for representative conjugates 9b are shown in Fig. S2.† Another interesting aspect of inhibition of tubulin polymerization is that different zinc-binding group and the linker length of HDACi unit do not affect the activity of conjugates. These experiment results showed that introduction of the HDACi unit onto levofloxacin retained great activity of their parent levofloxacin inhibited tubulin polymerization.

### Docking results

**Fig. 2:**
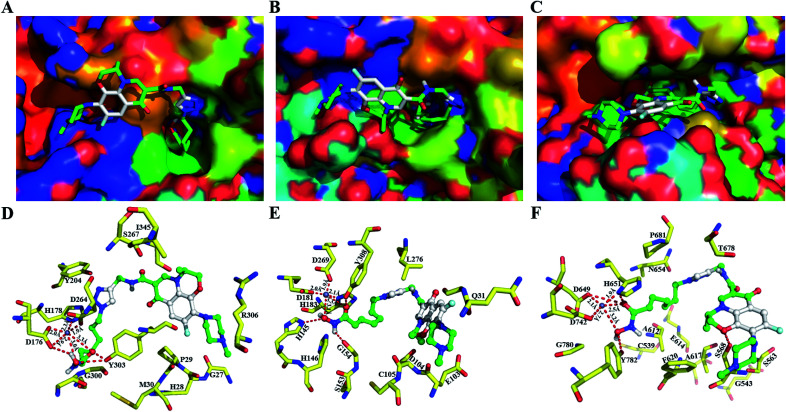
The predicted binding modes of conjugate 9b–HDAC1, HDAC2 and HDAC6. Molecular surface of the HDAC1 (A), HDAC2 (B), HDAC6 (C) binding pocket with docked conjugate 9b. (D) Docking poses of HDAC1–9b, which can form hydrogen bonds with residues D176, Y303 and which can coordinate the zinc ion with residues D181, H183, D269. (E) Docking poses of HDAC2–9b, which can form hydrogen bonds with residues H145, G154, Y308, and which can coordinate the zinc ion with residues D649, H651, D742. (F) Docking poses of HDAC6–9b, which can form hydrogen bonds with residues S568, D649, H651, Y782, and which can coordinate the zinc ion with residues D649, H651, D742. Distances are given in Å.

In HDACs inhibition activities, 9b exhibited the most potent anti-HDAC1 and HDAC6 activities. Therefore, to understand how conjugates 9b binds to these HDAC isoforms, the molecular docking study with conjugate 9b in HDAC1 (PDB: 4BKX),^36^ HDAC2 (PDB: 4LXZ)^37^ and HDAC6 (PDB: 5EDU)^38^ was performed, and the results are shown in Fig. 2.

Hydroxamic acid conjugate 9b adopts an optimal binding pose in both the catalytic domain of HDAC1, HDAC2 and HDAC6, and the surface recognition region levofloxacin unit of conjugate 9b occupies more cap region at the entrance of the HDAC binding pocket compared to phenyl group of SAHA (Fig. 2A–C). Therefore, the introduction of levofloxacin unit enhances the interaction of SAHA with HDAC1, HDAC2 and HDAC6. More importantly, the interaction of surface recognition region of HDACi with cap region of target proteins not only affected the inhibitory activities of HDACi, but also affected the selectivity of HDAC isoform.^39^ The oxygen atom of levofloxacin unit forms H-bonds with S568 in HDAC6 (Fig. 2F), while levofloxacin unit dose not forms similar H-bonds interaction in HDAC1, HDAC2 (Fig. 2D and E) which might explain the high HDAC6 selectivity of conjugate 9b. In addition, the hydroxamic tail of conjugate 9b forms key H-bonds with D176, and Y303 and coordinates with catalytic zinc ion (Zn^2+^–O of OH: 3.0 Å and Zn^2+^–O of CO: 3.3 Å) in active site of HDAC1, engages in key H-bonds with H145, G154, Y308 and coordinates with catalytic zinc ion (Zn^2+^–O of OH: 3.1 Å and Zn^2+^–O of CO: 3.3 Å) in active site of HDAC2, which also forms H-bonds with D649, H651, and Y782 and coordinates with catalytic zinc ion (Zn^2+^–O of OH: 3.2 Å and Zn^2+^–O of CO: 2.5 Å) in HDAC6. These hydrogen bond forces are critical in the catalytic mechanism as they stabilize HDAC inhibitor in a specific conformation chelating with zinc ion.^40,41^ This is consistent with the observed HDACs inhibition activities of conjugate 9b which exhibits excellent potency in inhibition for three isoforms HDAC1, HDAC2 and HDAC6.

**Fig. 3:**
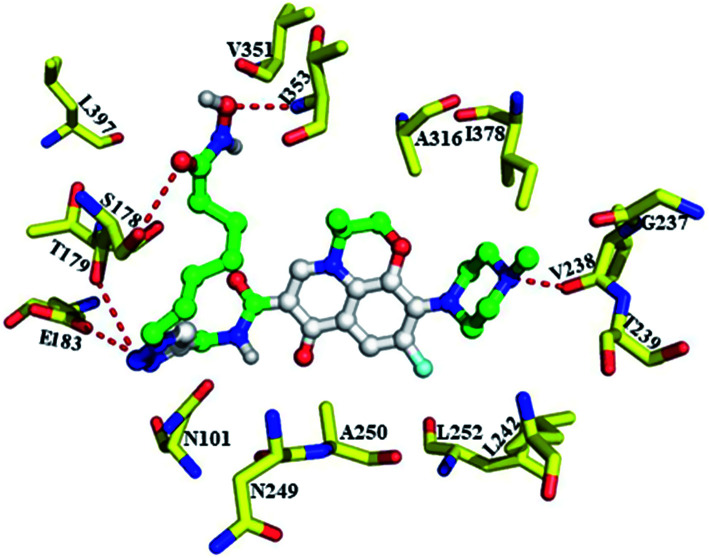
Predicted binding mode of 9b bound to the tubulin in the colchicine site with the conserved H-bond to S178, T179, E183, V238 and I353.

Our tubulin polymerization inhibition studies indicate that conjugate 9b bound to tubulin and inhibited polymerization. To further elucidate the binding characters of the 9b with tubulin, we also performed docking studies with 9b at the colchicine binding pocket (PDB: 4O2B).^42^ Docking investigation suggests that illustrates conjugate 9b overlaid with binding orientation of colchicine, and levofloxacin unit, hydroxamic motif and triazole linker of 9b all made hydrogen bonding interaction with residue of colchicine binding site (S178, T179, E183, V238 and I353, Fig. 3). These results further demonstrated that 9b possessed anti-tubulin properties.

### Whole cell antiproliferative activity

Conjugates 6–9 were evaluated for their *in vitro* antitumor activities against human lung adenocarcinoma cells A549, liver cancer cells Hepg2, breast cancer cells MCF-7, prostate cancer cells PC-3 and human cervical carcinoma cells HeLa by CCK-8 assay using levofloxacin and SAHA as the positive control, and the results are summarized in Table 3.

**Table 3:** Whole cell antiproliferative activity (IC_50_, μM)^a^

Compd	IC_50_ (μmol L^−1^)					
	A549	HepG2	MCF-7	PC-3	HeLa	MCF-10A
6a	14.2 ± 1.28	13.4 ± 1.36	10.3 ± 1.12	16.5 ± 1.63	12.3 ± 1.16	>100^b^
6b	10.7 ± 1.08	10.5 ± 1.17	8.9 ± 0.54	12.9 ± 1.25	9.7 ± 0.83	>100
6c	13.6 ± 1.16	12.7 ± 1.09	10.5 ± 1.22	15.8 ± 1.43	14.6 ± 1.08	>100
7a	12.8 ± 1.33	14.8 ± 1.27	9.7 ± 1.04	15.8 ± 1.48	13.5 ± 1.31	>100
7b	9.3 ± 0.82	9.9 ± 0.62	7.8 ± 0.71	11.1 ± 1.16	8.4 ± 0.62	>100
7c	11.9 ± 1.06	11.3 ± 1.24	12.7 ± 1.18	13.6 ± 1.29	10.6 ± 0.24	>100
8a	8.9 ± 0.72	9.8 ± 0.87	3.5 ± 0.51	12.8 ± 1.42	7.7 ± 0.64	>100
8b	3.5 ± 0.17	5.2 ± 0.56	0.8 ± 0.15	8.5 ± 0.79	2.7 ± 0.17	>100
8c	7.4 ± 0.44	5.7 ± 0.49	2.3 ± 0.27	10.4 ± 1.13	6.8 ± 0.56	>100
9a	5.6 ± 0.59	6.6 ± 0.78	1.1 ± 0.26	9.6 ± 1.05	5.2 ± 0.82	>100
9b	2.1 ± 0.26	2.3 ± 0.31	0.3 ± 0.14	4.9 ± 0.43	1.1 ± 0.15	>100
9c	4.2 ± 0.38	3.6 ± 0.55	1.1 ± 0.23	8.7 ± 0.62	3.5 ± 0.24	>100
SAHA	3.7 ± 0.23	3.6 ± 0.26	4.4 ± 0.35	6.4 ± 0.53	2.9 ± 0.18	25.2 ± 3.6
Levofloxacin	76.3 ± 6.51	>100	64.2 ± 5.67	>100	71.1 ± 4.98	>100

a IC_50_ values are an average of at least three independent experiments ± standard deviation (mean ± SD).

b IC_50_ not determinable up to highest concentrations tested.

Overall, the dual-acting conjugates 6–9 display potent inhibitory effect on the proliferation of these five cancer cells, A549, HepG2, MCF-7, PC-3, HeLa, and which is more effective compared with lead compound levofloxacin (IC_50_ > 70 μM); specifically, 9b stands out among these levofloxacin–HDACi conjugates because it shows the best antiproliferative activity, which is over 20-fold more potent than levofloxacin, against all cancer cells. The dose–response curves for antiproliferative activity of 9b and reference drug SelSA against MCF-7 cells are shown in Fig. S3.† This observation indicates that the antiproliferative activity of these conjugates derives mainly from the inhibition of HDAC, and inhibition of tubulin polymerization can enhance potency against cancer cells. It is worth mentioning that antiproliferative activities of these conjugates 6–9 are also very dependent on zinc-binding group and the linker length, and closely matched their anti-HDAC activities. We noticed that the general trend of conjugates against cancer cells still is that the hydroxamate conjugates 8–9 are more potent than their corresponding carboxylic acid conjugates 6–7; additionally, both carboxylic acid and hydroxamate conjugates with six methylene linker length have better inhibitory activities than the corresponding five or seven-methylene compounds (6b*vs.*6a and 6c, 7b*vs.*7a and 7c, 8b*vs.*8a and 8c, 9b*vs.*9a and 9c), this observation is in agreement with its HDAC inhibition profile. Moreover, hydroxamate conjugates 8–9 showed cell-type selectivity, which is selectively more potent against MCF-7 than those of A549, HepG2, PC-3, HeLa cells; yet, SAHA showed low cell-type selectivity. Additionally, all levofloxacin–HDACi conjugates are nontoxic to healthy MCF-10A cells, while SAHA showed considerable toxicity.

## Conclusions

In conclusion, we have successfully designed and synthesized a series of dual-action conjugates to target HADC and tubulin polymerization by having the hydroxamic acid group for chelation with zinc ion in the active site of HDAC and the key structural elements of levofloxacin unit for inhibition of tubulin polymerization. Careful analysis of their HDAC inhibition activity output shows hydroxamic acid conjugates 8–9 have superior HDAC inhibition activity relative to the carboxylic acid conjugates 6–7; moreover, linker length of HDACi unit is a critical factor for activity, and conjugates with the linker length of 6 carbons showed greatest HDAC inhibition activity, a shorter (*n* = 5) or longer (*n* = 7) chain length tend to decreased HDAC inhibition. The most potent conjugate 9b (*n* = 6) is at least 1.5-fold more potent than approved drug SAHA in inhibition for HDAC1 and HDAC6. Docking analyses of levofloxacin–HDACi conjugates also reveals levofloxacin unit of conjugate occupies more cap region than phenyl group of SAHA and enhances the interaction of SAHA with HDAC1, HDAC2 and HDAC6. Meanwhile, these conjugates retain great activity of their parent compounds in inhibiting tubulin polymerization. Furthermore, levofloxacin–HDACi conjugates exhibited superior antitumor potency and cell-type selectivity against breast cancer MCF-7 cells, and all conjugates are nontoxic to healthy VERO cells, while SAHA showed some extent inherent toxicity. These results indicate that conjugation of a HDACi moiety to representative quinolone derivative is a viable approach to generate dual action inhibitors which retain independent HDAC and tubulin polymerization inhibitory activities and have an improved therapeutic activity for cancer therapy.

## Experimental section

### General

Melting points were measured on an electrothermal melting point apparatus and are uncorrected. ^1^H NMR spectra were recorded in CDCl_3_ or acetone-*d*_6_ as solvent on a Bruker AVANCE-III 400 spectrometer and resonances are given in ppm relative to TMS. All of the solvents and materials were reagent grade and purified as required. All compounds were routinely checked by thin-layer chromatography (TLC) on pre-coated silica gel GF_254_ plates (Qingdao Haiyang Chemical Co., Ltd., P. R. China). Column chromatography was performed using silica gel (200–300 mesh) from Qingdao Haiyang Chemical Group Co., China.

#### (*S*)-Prop-2-yn-1-yl-9-fluoro-3-methyl-10-(4-methylpiperazin-1-yl)-7-oxo-3,7-dihydro-2*H*-[1,4]oxazino[2,3,4-*ij*]quinoline-6-carboxylate (2)

To a solution of levofloxacin (2.569 g, 7.1 mmol) in DMF (30 mL) was added propargyl bromo (1.855 g, 15.6 mmol) and NaHCO_3_ (1.310 g, 15.6 mmol). The reaction mixture was allowed to react at 100 °C for 48 h under argon. After completion of reaction, the solvent was removed under vacuum. The crude residue was purified by silica gel column chromatography with dichloromethane–methanol (10 : 1) to afford 2 as a white solid (1.670 g, 58.9%). Mp 178–181 °C; ^1^H NMR (CDCl_3_, 400 MHz): *δ* 8.86 (s, 1H), 7.88 (d, *J* = 13.2 Hz, 1H), 4.92 (d, *J* = 2.4 Hz, 2H), 4.67–4.54 (m, 3H), 3.37–3.24 (m, 4H), 2.65–2.54 (m, 4H), 2.42 (t, *J* = 2.8 Hz, 1H), 2.33 (s, 3H), 1.66 (d, *J* = 7.2 Hz, 3H).

#### (*S*)-9-Fluoro-3-methyl-10-(4-methylpiperazin-1-yl)-7-oxo-*N*-(prop-2-yn-1-yl)-3,7-dihydro-2*H*-[1,4]oxazino[2,3,4-*ij*]quinoline-6-carboxamide (3)

To a solution of levofloxacin (2.732 g, 7.5 mmol) in THF (30 mL) was added TBTU (3.628 g, 11.3 mmol) and DIPEA (2.908 g, 22.5 mmol). After the reaction mixture was stirred for 3 h at room temperature under argon, propargylamine (463 mg, 8.4 mmol) and K_2_CO_3_ (1.354 g, 9.8 mmol) were added. The reaction mixture was stirred for a further 1 h at room temperature, diluted with water (100 mL) and then extracted with EtOAc (3 × 30 mL). The extracts were dried (Na_2_SO_4_) and evaporated. The residue was purified by column chromatography (silica gel, petroleum ether–EtOAc, 8 : 2) to give 3 as a white solid (2.349 g, 90.7%). Mp 166–16 °C; ^1^H NMR (CDCl_3_, 400 MHz): *δ* 8.88 (s, 1H), 7.85 (d, *J* = 13.2 Hz, 1H), 6.15 (t, *J* = 4.4 Hz, 1H), 4.65–4.52 (m, 3H), 3.94–4.10 (m, 2H), 3.37–3.25 (m, 4H), 2.63–2.56 (m, 4H), 2.35 (s, 3H), 2.19 (t, *J* = 2.4 Hz, 1H), 1.65 (d, *J* = 7.2 Hz, 3H).

#### General procedure for azido compounds (5a–c)

A mixture of compound 4 (6.8 mmol) with NaN_3_ (1.326 g, 20.4 mmol) and DMF (15 mL) was stirred at 77 °C for 30 h, diluted with the mixture of petroleum ether and EtOAc (50 mL, 4 : 1, v/v), and extracts were washed with saturated NaHCO_3_ (3 × 20 mL) and water (3 × 30 mL), dried (Na_2_SO_4_), and concentrated under reduced pressure. The residue was purified by column chromatography (silica gel, petroleum ether–EtOAc, 1 : 1) to give 5.

##### 6-Azidohexanoic acid (5a)

White oil, 89.7% yield; ^1^H NMR (CDCl_3_, 400 MHz): *δ* 3.32–3.39 (m, 2H), 2.43 (t, *J* = 7.2 Hz, 2H), 1.72–1.89 (m, 2H), 1.54–1.66 (m, 2H), 1.33–1.41 (m, 2H).

##### 7-Azidoheptanoic acid (5b)

White oil, 92.5% yield; ^1^H NMR (CDCl_3_, 400 MHz): *δ* 3.31 (t, *J* = 6.8 Hz, 2H), 2.42 (t, *J* = 6.8 Hz, 2H), 1.73–1.90 (m, 2H), 1.58–1.69 (m, 2H), 1.36–1.53 (m, 4H).

##### 8-Azidooctanoic acid (5d)

White oil, 91.3% yield; ^1^H NMR (CDCl_3_, 400 MHz): *δ* 3.32 (t, *J* = 6.8 Hz, 2H), 2.43 (t, *J* = 7.2 Hz, 2H), 1.72–1.91 (m, 2H), 1.55–1.67 (m, 2H), 1.31–1.52 (m, 6H).

#### General procedure for carboxylic acid conjugates (6–7)

To a solution of compound 2 or 3 (1.8 mmol) in a mixed solvent (5 mL THF and 5 mL H_2_O), azido compounds 5 (3.6 mmol), CuSO_4_ (64 mg, 0.4 mmol) and l-sodium ascorbate (139 mg, 0.7 mmol) were added. The reaction solution was stirred at room temperature for 20 h under argon, diluted with water (100 mL) and then extracted with DCM (3 × 25 mL). The extracts were dried (Na_2_SO_4_) and evaporated. The residue was purified by column chromatography (silica gel, petroleum ether–EtOAc, 8 : 2) to give carboxylic acid conjugates 6–7.

##### (*S*)-6-(4-(((9-fluoro-3-methyl-10-(4-methylpiperazin-1-yl)-7-oxo-3,7-dihydro-2*H*-[1,4]oxazino[2,3,4-*ij*]quinoline-6-carbonyl)oxy)methyl)-1*H*-1,2,3-triazol-1-yl)hexanoic acid (6a)

White solid, yield 69.2%, mp 175–178 °C; ^1^H NMR (400 MHz, acetone-*d*_6_): *δ* 8.84 (s, 1H), 7.97 (s, 1H), 7.86 (d, *J* = 13.2 Hz, 1H), 5.51 (s, 2H), 4.65–4.53 (m, 3H), 4.35 (t, *J* = 7.2 Hz, 2H), 3.39–3.25 (m, 4H), 2.68–2.59 (m, 4H), 2.32 (s, 3H), 1.95 (t, *J* = 7.2 Hz, 2H), 1.71–1.67 (m, 5H), 1.63 (t, *J* = 7.6 Hz, 2H), 1.35–1.29 (m, 2H). HRMS (ESI) calcd for C_28_H_33_FN_5_O_6_ [M − H]^−^, 554.5994; found 554.5988.

##### (*S*)-7-(4-(((9-fluoro-3-methyl-10-(4-methylpiperazin-1-yl)-7-oxo-3,7-dihydro-2*H*-[1,4]oxazino[2,3,4-*ij*]quinoline-6-carbonyl)oxy)methyl)-1*H*-1,2,3-triazol-1-yl)heptanoic acid (6b)

White solid, yield 63.4%, mp 183–185 °C; ^1^H NMR (400 MHz, acetone-*d*_6_): *δ* 8.86 (s, 1H), 7.94 (s, 1H), 7.87 (d, *J* = 13.2 Hz, 1H), 5.51 (s, 2H), 4.67–4.58 (m, 3H), 4.34 (t, *J* = 6.8 Hz, 2H), 3.36–3.24 (m, 4H), 2.77–2.54 (m, 4H), 2.37 (s, 3H), 1.95–1.85 (m, 2H), 1.83–1.71 (m, 2H), 1.69–1.55 (m, 5H), 1.42–1.36 (m, 2H), 1.35–1.24 (m, 2H). HRMS (ESI) calcd for C_29_H_35_FN_5_O_6_ [M − H]^−^, 568.6264; found 568.6257.

##### (*S*)-8-(4-(((9-fluoro-3-methyl-10-(4-methylpiperazin-1-yl)-7-oxo-3,7-dihydro-2*H*-[1,4]oxazino[2,3,4-*ij*]quinoline-6-carbonyl)oxy)methyl)-1*H*-1,2,3-triazol-1-yl)octanoic acid (6c)

White solid, yield 70.5%, mp 196–199 °C; ^1^H NMR (400 MHz, acetone-*d*_6_): *δ* 8.86 (s, 1H), 7.95 (s, 1H), 7.88 (d, *J* = 13.6 Hz, 1H), 5.48 (s, 2H), 4.68–4.52 (m, 3H), 4.32 (t, *J* = 7.2 Hz, 2H), 3.38–3.30 (m, 4H), 2.69–2.56 (m, 4H), 2.34 (s, 3H), 1.98–1.87 (m, 2H), 1.83 (t, *J* = 6.8 Hz, 2H), 1.68–1.55 (m, 5H), 1.43–1.31 (m, 2H), 1.22–1.19 (m, 4H). HRMS (ESI) calcd for C_30_H_37_FN_5_O_6_ [M − H]^−^, 582.6534; found 582.6530.

##### (*S*)-6-(4-((9-fluoro-3-methyl-10-(4-methylpiperazin-1-yl)-7-oxo-3,7-dihydro-2*H*-[1,4]oxazino[2,3,4-*ij*]quinoline-6-carboxamido)methyl)-1*H*-1,2,3-triazol-1-yl)hexanoic acid (7a)

White solid, yield 68.1%, mp 179–182 °C; ^1^H NMR (400 MHz, acetone-*d*_6_): *δ* 8.84 (s, 1H), 7.99 (s, 1H), 7.88 (d, *J* = 13.6 Hz, 1H), 6.62 (t, *J* = 4.8 Hz, 1H), 5.42 (t, *J* = 15.2 Hz, 2H), 4.65–4.53 (m, 3H), 4.34 (t, *J* = 7.6 Hz, 2H), 3.37–3.32 (m, 4H), 2.67–2.61 (m, 4H), 2.38 (s, 3H), 1.97–1.89 (m, 2H), 1.74–1.65 (m, 5H), 1.63–1.56 (m, 2H), 1.32–1.25 (m, 2H). HRMS (ESI) calcd for C_28_H_34_FN_6_O_5_ [M − H]^−^, 553.6154; found 553.6150.

##### (*S*)-7-(4-((9-fluoro-3-methyl-10-(4-methylpiperazin-1-yl)-7-oxo-3,7-dihydro-2*H*-[1,4]oxazino[2,3,4-*ij*]quinoline-6-carboxamido)methyl)-1*H*-1,2,3-triazol-1-yl)heptanoic acid (7b)

White solid, yield 72.4%, mp 187–190 °C; ^1^H NMR (400 MHz, acetone-*d*_6_): *δ* 8.88 (s, 1H), 7.93 (s, 1H), 7.86 (d, *J* = 13.2 Hz, 1H), 6.63 (t, *J* = 4.8 Hz, 1H), 5.48 (t, *J* = 14.8 Hz, 2H), 4.67–4.55 (m, 3H), 4.34–4.26 (m, 2H), 3.37–3.22 (m, 4H), 2.68–2.56 (m, 4H), 2.35 (s, 3H), 1.94 (t, *J* = 6.8 Hz, 2H), 1.82–1.73 (m, 2H), 1.69 (d, *J* = 7.2 Hz, 3H), 1.63–1.53 (m, 2H), 1.39–1.26 (m, 4H). HRMS (ESI) calcd for C_29_H_36_FN_6_O_5_ [M − H]^−^, 567.6424; found 567.6418.

##### (*S*)-8-(4-((9-fluoro-3-methyl-10-(4-methylpiperazin-1-yl)-7-oxo-3,7-dihydro-2*H*-[1,4]oxazino[2,3,4-*ij*]quinoline-6-carboxamido)methyl)-1*H*-1,2,3-triazol-1-yl)octanoic acid (7c)

White solid, yield 67.7%, mp 203–205 °C; ^1^H NMR (400 MHz, acetone-*d*_6_): *δ* 8.87 (s, 1H), 7.94 (s, 1H), 7.88 (d, *J* = 13.2 Hz, 1H), 6.64 (t, *J* = 4.4 Hz, 1H), 5.41 (t, *J* = 15.2 Hz, 2H), 4.63–4.57 (m, 3H), 4.31–4.23 (m, 2H), 3.38–3.27 (m, 4H), 2.66–2.55 (m, 4H), 2.33 (s, 3H), 1.94 (t, *J* = 6.8 Hz, 2H), 1.79 (t, *J* = 7.2 Hz, 2H), 1.64 (d, *J* = 6.8 Hz, 3H), 1.63–1.51 (m, 6H), 1.40–1.21 (m, 2H). HRMS (ESI) calcd for C_30_H_38_FN_6_O_5_ [M − H]^−^, 581.6694; found 581.6689.

#### General procedure for hydroxamic acid (8–9)

To a 0 °C cooled solution of carboxylic acid conjugates 6 or 7 (1.5 mmol) in dry THF (10 mL), ClCO_2_Et (239 mg, 2.2 mmol) and Et_3_N (304 mg, 3.0 mmol) were added, and the mixture was stirred for 15 min under argon. After that, the freshly prepared solution of hydoxylamine in methanol was added. To prepare the hydroxylamine, a solution of hydroxylamine hydrochloride (306 mg, 4.4 mmol) in methanol (10 mL), KOH (252 mg, 4.5 mmol) was added at 40 °C for 15 min. The reaction mixture was cooled to 0 °C, the precipitate was filtered off, and the filtrate was used as fresh hydoxylamine solution. The resulting mixture was stirred for 1 h and then was evaporated, and the residue was purified by silica gel column chromatography (dichlormethane–MeOH, 15 : 1) to give hydroxamic acid conjugates 8 or 9.

##### (*S*)-(1-(6-(hydroxyamino)-6-oxohexyl)-1*H*-1,2,3-triazol-4-yl)methyl-9-fluoro-3-methyl-10-(4-methylpiperazin-1-yl)-7-oxo-3,7-dihydro-2*H*-[1,4]oxazino[2,3,4-*ij*]quinoline-6-carboxylate (8a)

White solid, yield 77.6%, mp 189–192 °C; ^1^H NMR (400 MHz, acetone-*d*_6_): *δ* 9.48 (s, 1H), 8.86 (s, 1H), 8.01 (s, 1H), 7.87 (d, *J* = 13.2 Hz, 1H), 5.49 (s, 2H), 4.67–4.56 (m, 3H), 4.39 (t, *J* = 6.8 Hz, 2H), 3.37–3.25 (m, 4H), 2.67–2.56 (m, 4H), 2.41–2.36 (m, 2H), 2.35 (s, 3H), 1.62–1.47 (m, 5H), 1.39–1.32 (m, 2H), 1.31–1.25 (m, 2H). HRMS (ESI) calcd for C_28_H_36_FN_6_O_6_ [M − H]^−^, 571.6304; found 571.6310.

##### (*S*)-(1-(7-(hydroxyamino)-7-oxoheptyl)-1*H*-1,2,3-triazol-4-yl)methyl-9-fluoro-3-methyl-10-(4-methylpiperazin-1-yl)-7-oxo-3,7-dihydro-2*H*-[1,4]oxazino[2,3,4-*ij*]quinoline-6-carboxylate (8b)

White solid, yield 71.9%, mp 211–214 °C; ^1^H NMR (400 MHz, acetone-*d*_6_): *δ* 9.73 (s, 1H), 8.87 (s, 1H), 7.96 (s, 1H), 7.86 (d, *J* = 12.8 Hz, 1H), 5.52 (s, 2H), 4.68–4.55 (m, 3H), 4.36 (t, *J* = 7.2 Hz, 2H), 3.35–3.22 (m, 4H), 2.67–2.55 (m, 4H), 2.46 (t, *J* = 7.2 Hz, 2H), 2.38 (s, 3H), 1.87–1.76 (m, 2H), 1.69 (d, *J* = 7.6 Hz, 3H), 1.57–1.46 (m, 2H), 1.39–1.22 (m, 4H). HRMS (ESI) calcd for C_29_H_38_FN_6_O_6_ [M − H]^−^, 585.6574; found 585.6582.

##### (*S*)-(1-(8-(hydroxyamino)-8-oxooctyl)-1*H*-1,2,3-triazol-4-yl)methyl-9-fluoro-3-methyl-10-(4-methylpiperazin-1-yl)-7-oxo-3,7-dihydro-2*H*-[1,4]oxazino[2,3,4-*ij*]quinoline-6-carboxylate (8c)

White solid, yield 73.8%, mp 225–228 °C; ^1^H NMR (400 MHz, acetone-*d*_6_): *δ* 9.67 (s, 1H), 8.89 (s, 1H), 7.97 (s, 1H), 7.88 (d, *J* = 13.2 Hz, 1H), 5.49 (s, 2H), 4.65–4.54 (m, 3H), 4.33 (t, *J* = 6.8 Hz, 2H), 3.37–3.24 (m, 4H), 2.65–2.57 (m, 4H), 2.48–2.40 (m, 2H), 2.37 (s, 3H), 1.85–1.78 (m, 2H), 1.67 (d, *J* = 6.4 Hz, 3H), 1.64–1.57 (m, 2H), 1.41–1.32 (m, 4H), 1.31–1.25 (m, 2H). HRMS (ESI) calcd for C_30_H_40_FN_6_O_6_ [M − H]^−^, 599.6844; found 599.6848.

##### (*S*)-9-Fluoro-*N*-((1-(6-(hydroxyamino)-6-oxohexyl)-1*H*-1,2,3-triazol-4-yl)methyl)-3-methyl-10-(4-methylpiperazin-1-yl)-7-oxo-3,7-dihydro-2*H*-[1,4]oxazino[2,3,4-*ij*]quinoline-6-carboxamide (9a)

White solid, yield 69.2%, mp 202–205 °C; ^1^H NMR (400 MHz, acetone-*d*_6_): *δ* 9.74 (s, 1H), 8.89 (s, 1H), 7.98 (s, 1H), 7.87 (d, *J* = 13.2 Hz, 1H), 6.60 (t, *J* = 4.4 Hz, 1H), 5.47 (t, *J* = 14.8 Hz, 2H), 4.68–4.57 (m, 3H), 4.34–4.26 (m, 2H), 3.37–3.22 (m, 4H), 2.71–2.64 (m, 4H), 2.42 (t, *J* = 6.8 Hz, 2H), 2.35 (s, 3H), 1.72–1.68 (m, 2H), 1.65 (t, *J* = 6.4 Hz, 2H), 1.62–1.54 (m, 2H), 1.31–1.24 (m, 2H). HRMS (ESI) calcd for C_28_H_37_FN_7_O_5_ [M − H]^−^, 570.6464; found 570.6470.

##### (*S*)-9-Fluoro-*N*-((1-(7-(hydroxyamino)-7-oxoheptyl)-1*H*-1,2,3-triazol-4-yl)methyl)-3-methyl-10-(4methylpiperazin-1-yl)-7-oxo-3,7-dihydro-2*H*-[1,4]oxazino[2,3,4-*ij*]quinoline-6-carboxamide (9b)

White solid, yield 67.3%, mp 222–225 °C; ^1^H NMR (400 MHz, acetone-*d*_6_): *δ* 9.71 (s, 1H), 8.89 (s, 1H), 7.95 (s, 1H), 7.89 (d, *J* = 13.6 Hz, 1H), 6.62 (t, *J* = 4.4 Hz, 1H), 5.51 (t, *J* = 15.2 Hz, 2H), 4.67–4.58 (m, 3H), 4.34 (t, *J* = 6.8 Hz, 2H), 3.36–3.29 (m, 4H), 2.68–2.57 (m, 4H), 2.46–2.41 (m, 2H), 2.33 (s, 3H), 1.96 (t, *J* = 6.8 Hz, 2H), 1.67 (d, *J* = 7.2 Hz, 3H), 1.57–1.52 (m, 2H),1.33–1.22 (m, 4H). HRMS (ESI) calcd for C_29_H_39_FN_7_O_5_ [M − H]^−^, 584.6734; found 584.6739.

##### (*S*)-9-Fluoro-*N*-((1-(8-(hydroxyamino)-8-oxooctyl)-1*H*-1,2,3-triazol-4-yl)methyl)-3-methyl-10-(4-methylpiperazin-1-yl)-7-oxo-3,7-dihydro-2*H*-[1,4]oxazino[2,3,4-*ij*]quinoline-6-carboxamide (9c)

White solid, yield 72.8%, mp 231–234 °C; ^1^H NMR (400 MHz, acetone-*d*_6_): *δ* 9.75 (s, 1H), 8.88 (s, 1H), 7.99 (s, 1H), 7.86 (d, *J* = 13.6 Hz, 1H), 6.62 (t, *J* = 4.8 Hz, 1H), 5.52 (t, *J* = 15.6 Hz, 2H), 4.67–4.53 (m, 3H), 4.32 (t, *J* = 7.2 Hz, 2H), 3.40–3.22 (m, 4H), 2.69–2.58 (m, 4H), 2.46–2.41 (m, 2H), 2.36 (s, 3H), 1.94 (t, *J* = 6.8 Hz, 2H), 1.68 (d, *J* = 7.2 Hz, 3H), 1.65–1.51 (m, 2H), 1.37–1.23 (m, 6H). HRMS (ESI) calcd for C_30_H_41_FN_7_O_5_ [M − H]^−^, 598.7004; found 598.7011.

### HDAC activity assay

HDAC activity was measured using fluorogenic HDACs assay kit (BPS Bioscience, CA) according to the manufacturer's protocol. The enzymatic reactions were conducted in duplicate at 37 °C for 30 min in a mixture containing HDAC enzyme (human recombinant HDAC1, HDAC2 and HDAC6), BSA, HDAC substrate, HDAC assay buffer and various concentrations of tested compound. Fluorescence values were measured at an excitation of 350 nm and an emission of 440 nm using SpectraMax M2 microplate reader. IC_50_ values were calculated using Origin software. IC_50_ values were determined using nonlinear regression analysis.

### Tubulin polymerization assay

Tubulin polymerization assay was performed using a fluorescence-based tubulin polymerization assay Kit (BK011, Cytoskeleton, Inc.) according to the manufacturer's protocol. Assay mixtures containing 10 μM (1.2 mg mL^−1^) tubulin and varying concentrations of conjugates 6–9 were pre-incubated with guanosine 5′-triphosphate (GTP) for 15 min at 30 °C and then cooled to 0 °C. After addition of polymerization buffer and 0.4 mM GTP, the reaction mixtures were transferred to 0 °C cuvettes in a recording spectrophotometer. Reactions were followed at 37 °C over 60 min, monitoring OD_340_ at 1 min intervals. Data from each well was normalized relative to initial readings,^43^ and plots of ΔOD_max_ (final−initial values) against compound concentration, expressed relative to vehicle control (DMSO only), were used to calculate IC_50_ values.^44^ IC_50_ values were determined using nonlinear regression analysis.

## Molecular modeling

The crystal structure of HDAC1 (PDB: 4BKX),^36^ HDAC2 (PDB: 4LXZ),^37^ HDAC6 (PDB: 5EDU)^38^ and tubulin (PDB: 4O2B)^42^ were obtained from the PDB and all water molecules were removed. Crystallographic coordinate of the 9b was created by Biochemoffice. Preparations of all ligands and the protein were performed with AutoDockTools (ADT) and conjugate 9b was docked into the structure of HDAC1 and HDAC6 with AutoDock software (version 4.2). The figures were prepared using PyMOL.

### Antitumor activity

Antitumor activities *in vitro* of synthetic compounds were measured using CCK-8 Assay Kit (BPS Bioscience, CA) according to the manufacturer's protocol. Cells were plated into 96-well plates with appropriate test compounds for 72 hours. Control cells were treated with serum free medium. Then CCK-8 solution was added to each well to determine cell viability. The IC_50_ values were calculated according to the dose-dependent curves (Origin software). IC_50_ values were determined using nonlinear regression analysis.

## Conflicts of interest

There are no conflicts to declare.

## Supplementary Material

RA-008-C8RA02578A-s001
