# Supplementary material for: Synthesis and biological evaluation of novel quinolone derivatives dual targeting histone deacetylase and tubulin polymerization as antiproliferative agents
Source: RSC Adv. 2018 May 4;8(30):16494–502. doi: 10.1039/c8ra02578a (PMC9080233; doi:10.1039/c8ra02578a)

Electronic Supplementary Material (ESI) for RSC Advances.
This journal is © The Royal Society of Chemistry 2018

Supporting information for

Synthesis, biological evaluation of novel quinolone derivatives dual targeting histone deacetylase and tubulin polymerization as antiproliferative agents

Xuan Wang, Xiaoye Jiang, Shiyou Sun, Yongqiong Liu

City College, Wuhan University of Science and Technology, Wuhan 430000, China

Table of Contents

PART I. Analytical techniques……………………………………………….….S2

PART II. IC_{50} graphs of representative compound 9b..…………….............S3

PART III. ^{1}H NMR Spectra of final compounds…..……………..............S4-9

PART I. Analytical techniques

^{1}H NMR and ^{13}C NMR spectra were recorded on a Bruker AV400 spectrometer (400 MHz, ^{1}H NMR; 101 MHz, ^{13}C NMR) at room temperature. NMR spectra were calibrated to the solvent signals of $CDCl_3$ (δ 7.26 and 77.00), Acetone-d_6 (δ 2.05 and 29.84, 206.26). The chemical shifts are provided in ppm and the coupling constants in Hz. The following abbreviations for multiplicities are used: s, singlet; d, doublet; t, triplet; m, multiplet. Glassware was oven-dried, assembled while hot, and cooled under an inert atmosphere. Unless otherwise noted, all reactions were conducted in an inert atmosphere. Reaction progress was monitored using analytical thin-layer chromatography (TLC). Visualization was achieved by UV light (254 nm). Chromatography was performed with silica gel (0.040-0.063 mm) packing. High resolution mass spectra (HRMS) were measured on an IonSpec 4.7 Tesla FTMS using MALDI/DHB. Melting points were obtained on an X-4 melting point apparatus (Beijing TECH Instruments, Co.,Ltd.) and are uncorrected.

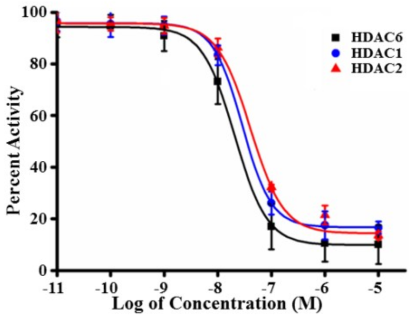

Figure S1 Dose-response curves and derived IC_{50} value for **9b** in HDACs inhibition assay.

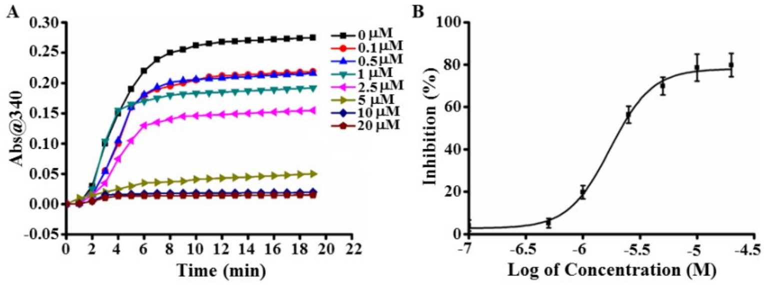

Figure S2 (A) Tubulin polymerization time-course plots following changes in OD_{340} of reaction mixtures in the presence of increasing concentrations of **9b**. (B) Dose-response curves and derived IC_{50} value for **9b** in the tubulin polymerization assay. Data from three independent experiments was plotted as individual data points, as shown, and fit globally.

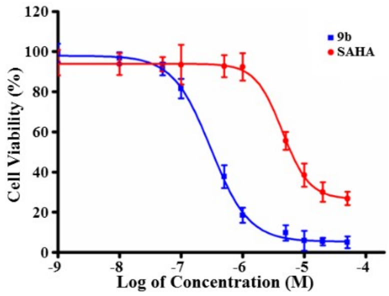

Figure S3 Dose-response curves for antiproliferative activity of **9b** and SAHA against MCF-7 cells.

PART II. ^{1}H NMR Spectra of final compounds

^{1}H NMR of compound 6a

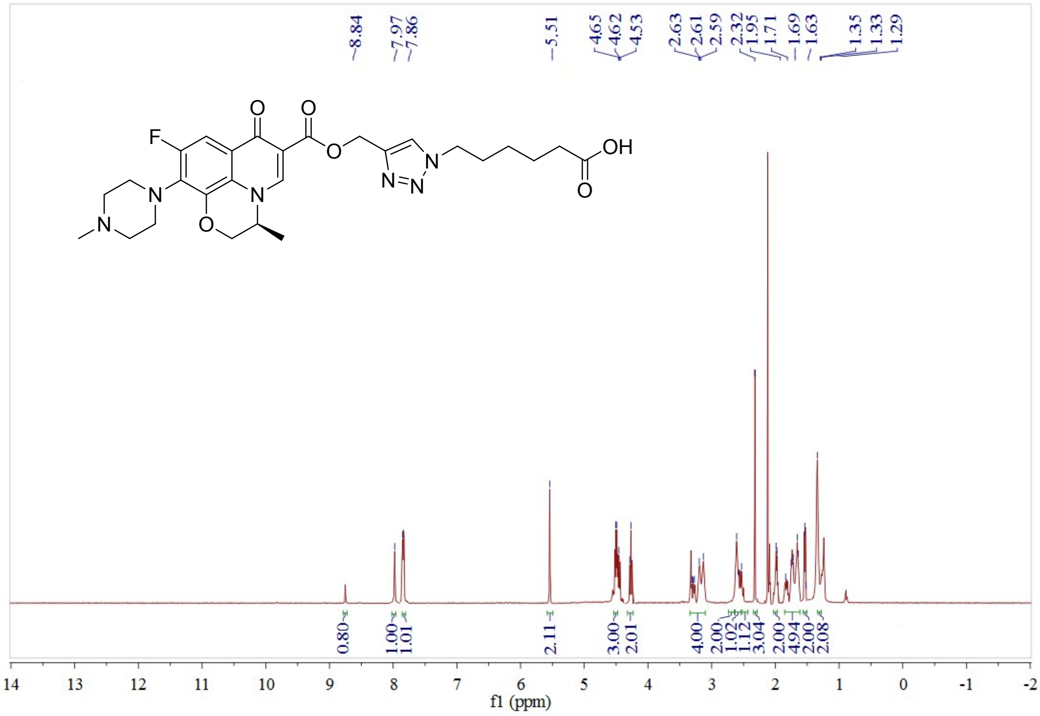

^{1}H NMR of compound 6b

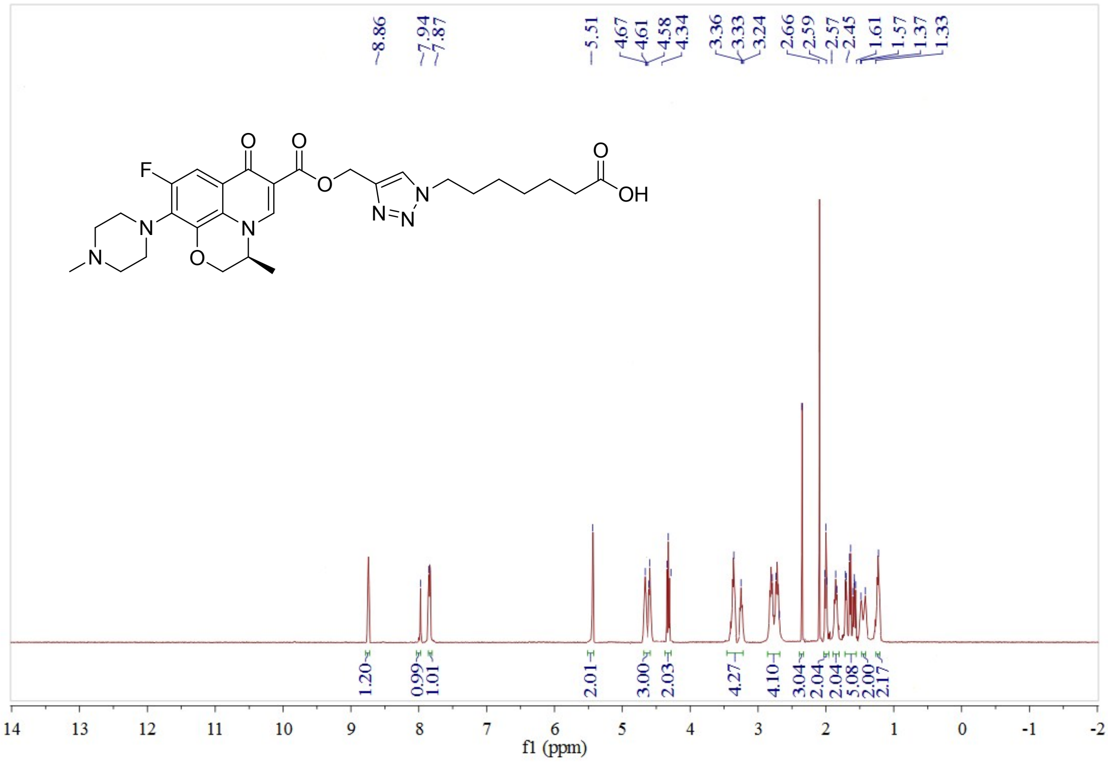

^{1}H NMR of compound 6c

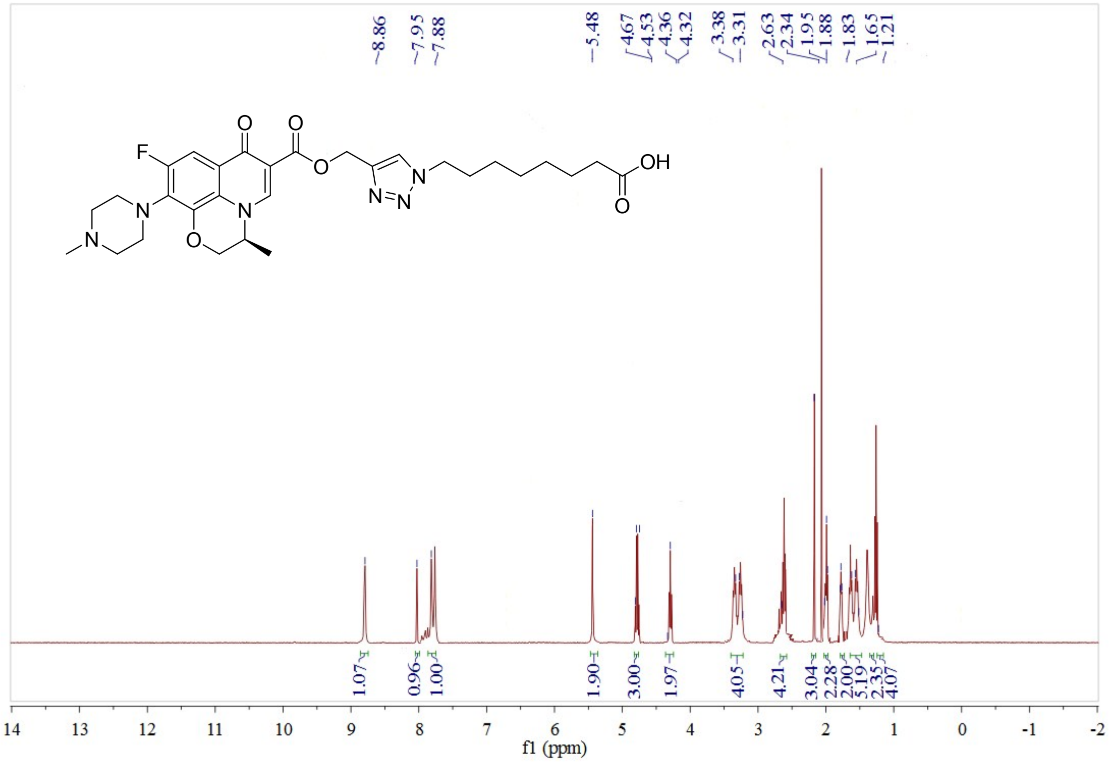

^{1}H NMR of compound 7a

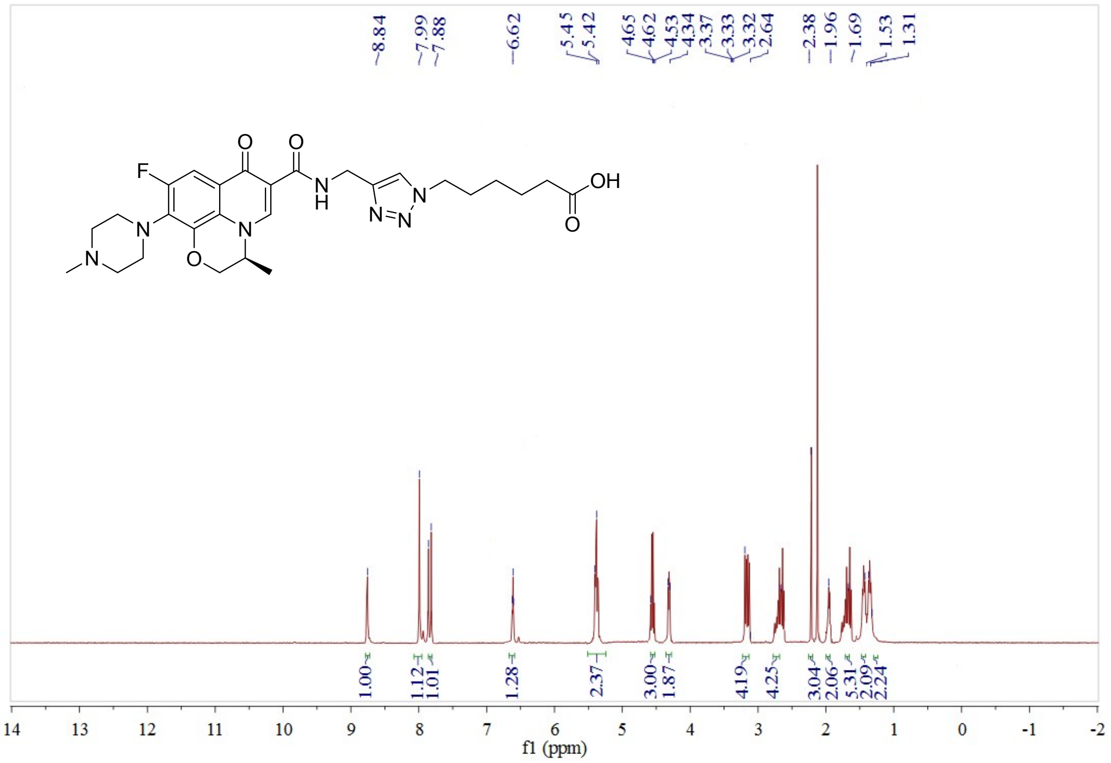

^{1}H NMR of compound 7b

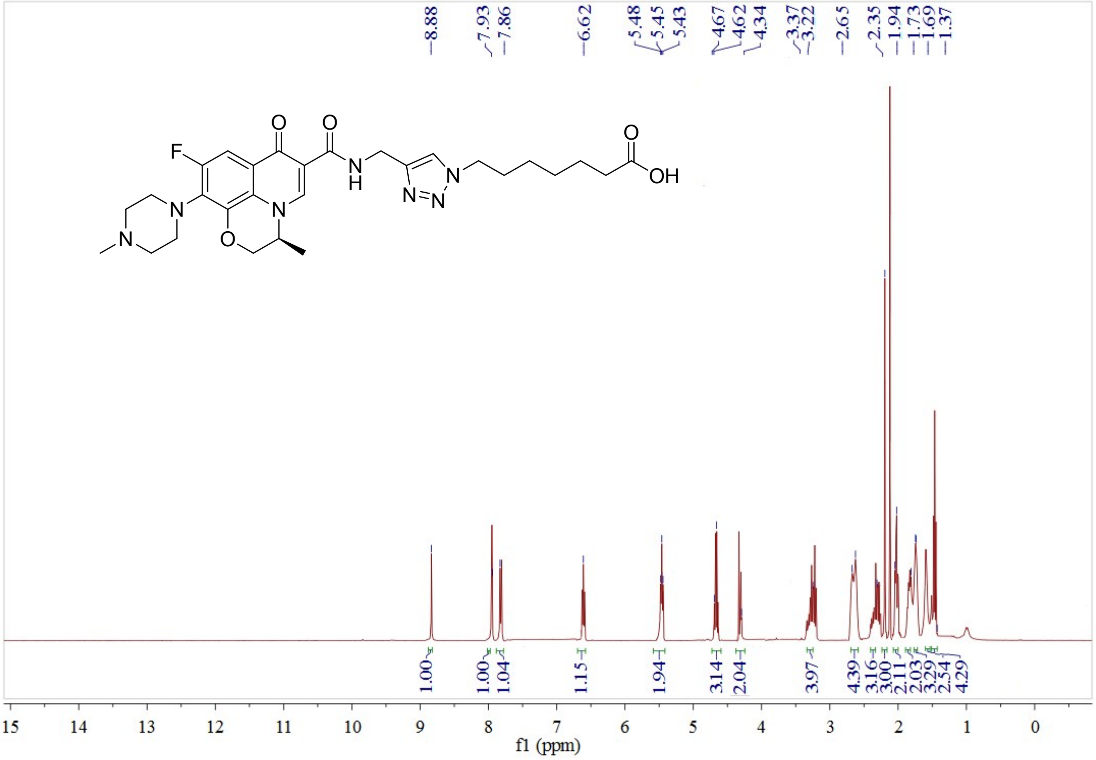

^{1}H NMR of compound 7c

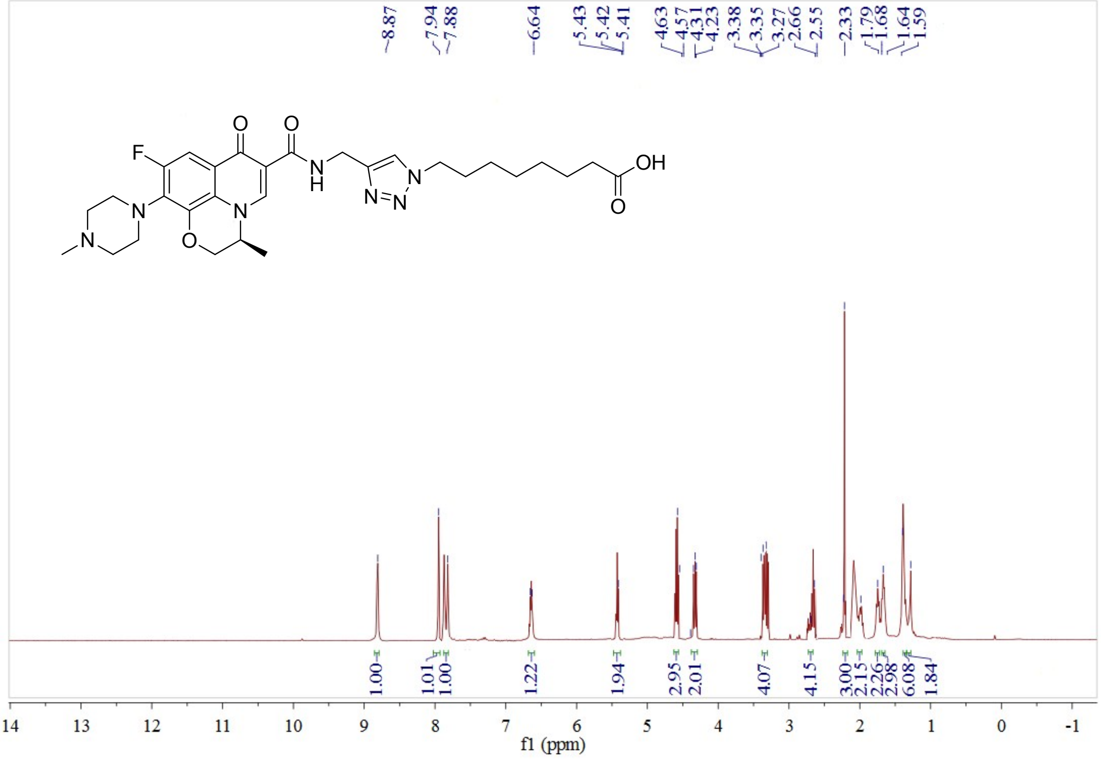

^{1}H NMR of compound 8a

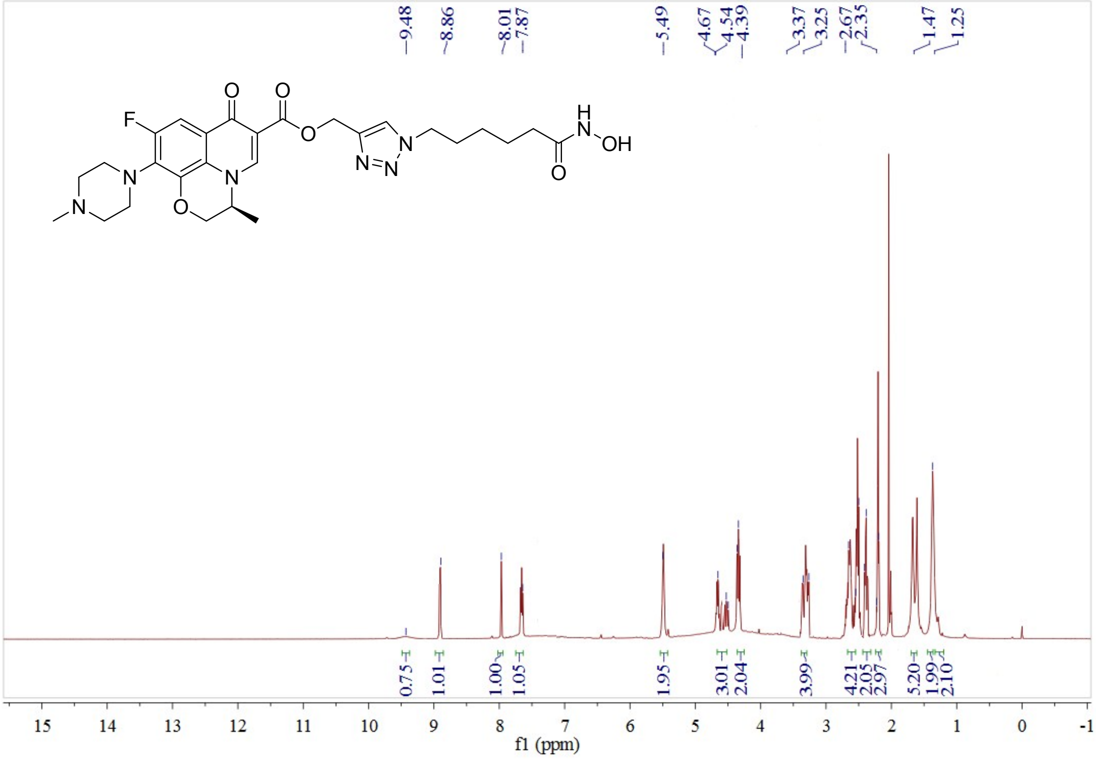

^{1}H NMR of compound 8b

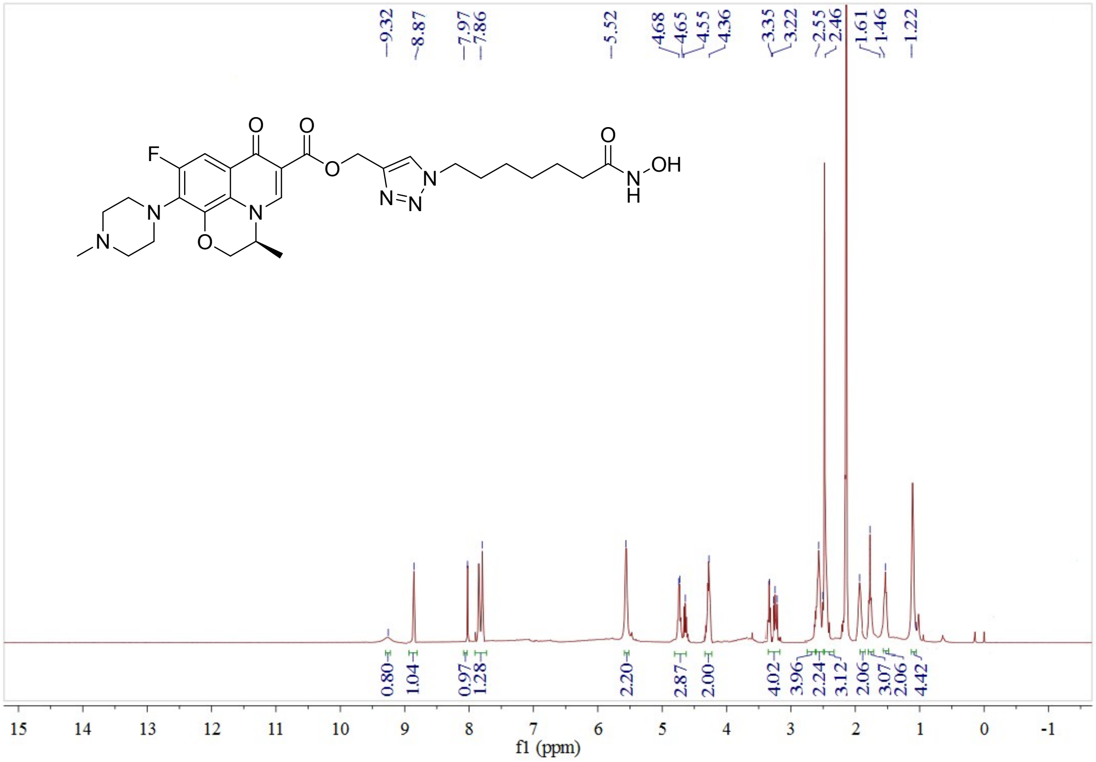

^{1}H NMR of compound 8c

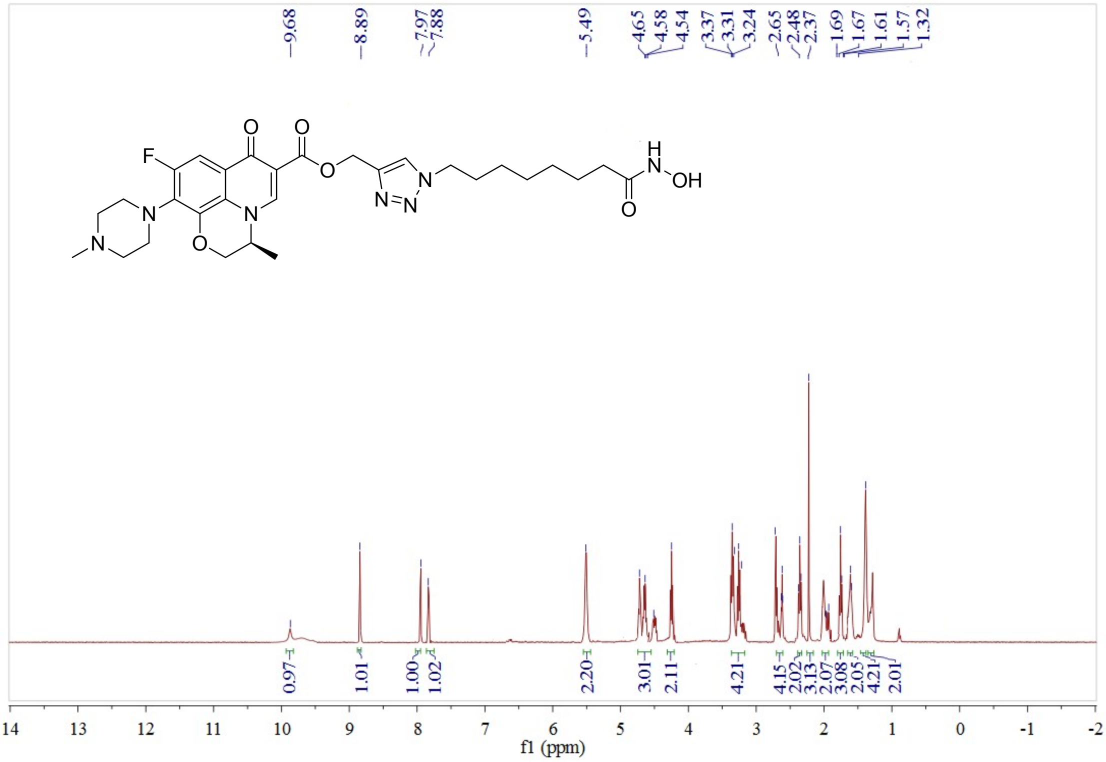

^{1}H NMR of compound 9a

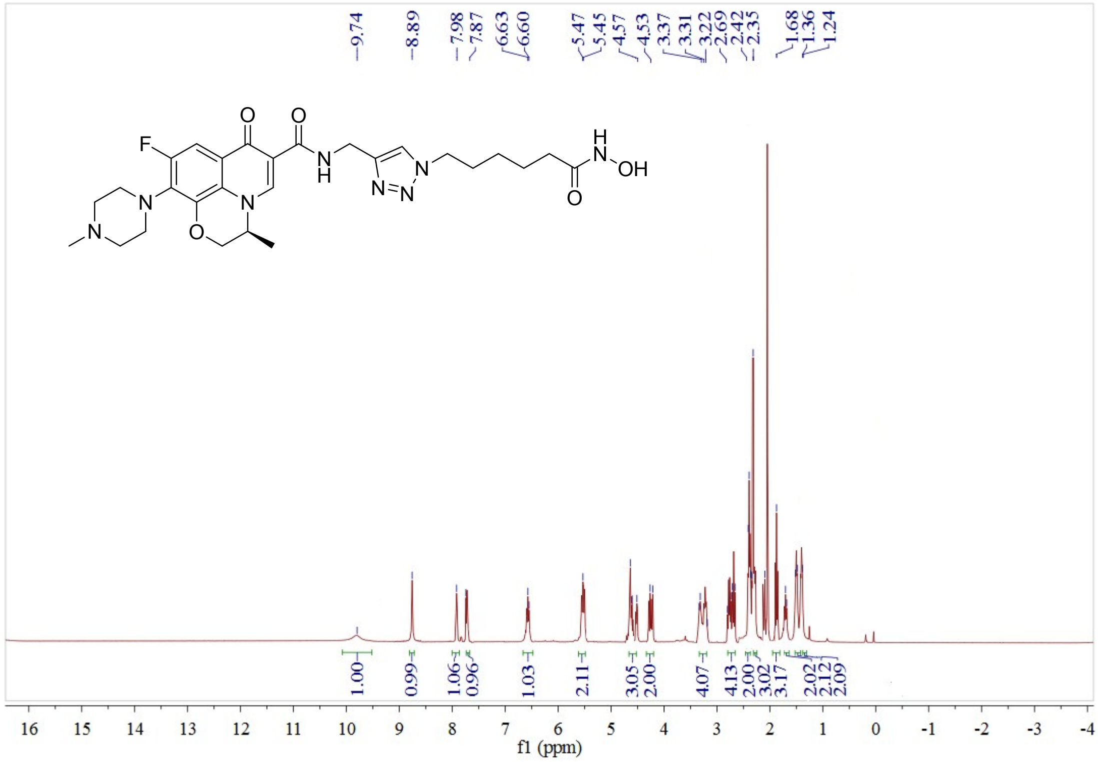

^{1}H NMR of compound 9b

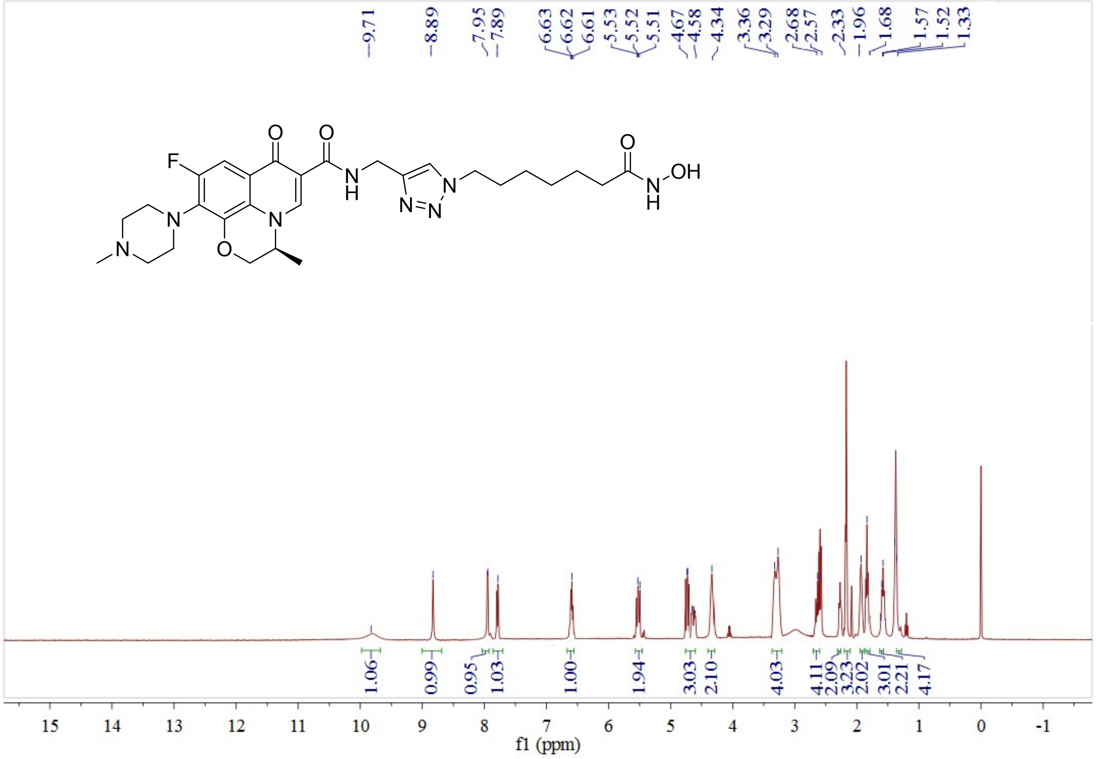

^{1}H NMR of compound 9c

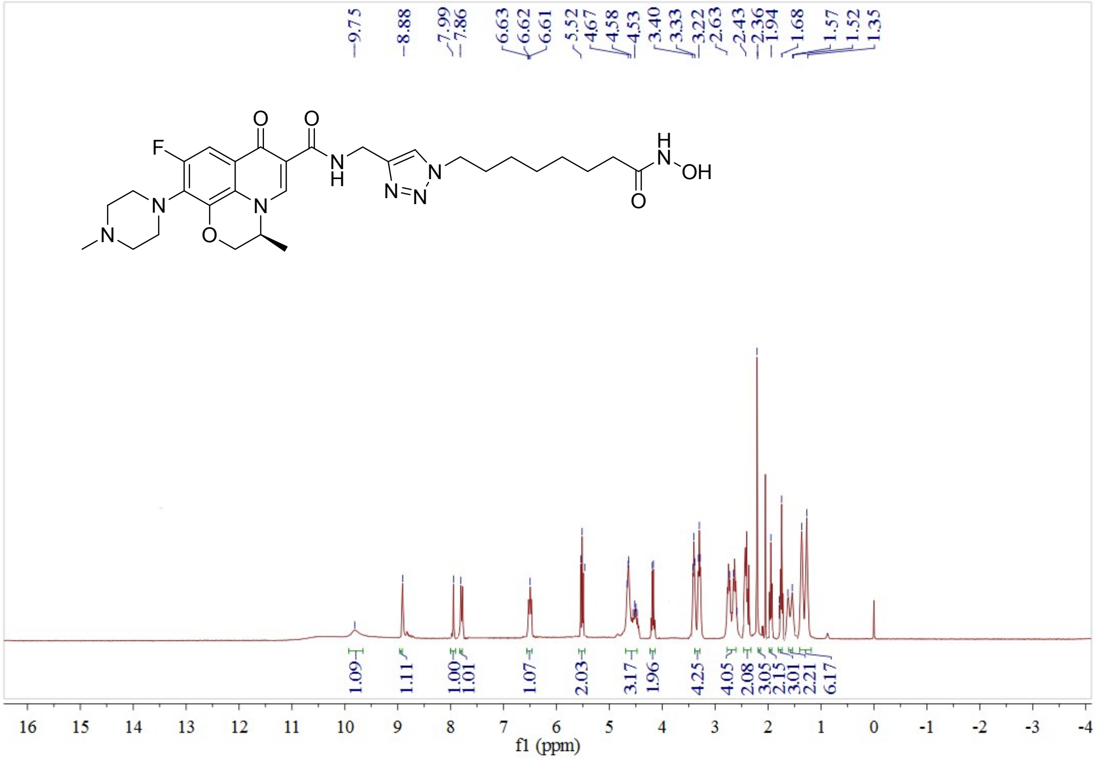

Supplement: RA-008-C8RA02578A-s001 [file RA-008-C8RA02578A-s001.pdf]
